# Low survival of strongly footed pheasants may explain constraints on lateralization

**DOI:** 10.1038/s41598-018-32066-1

**Published:** 2018-09-13

**Authors:** Mark A. Whiteside, Mackenzie M. Bess, Elisa Frasnelli, Christine E. Beardsworth, Ellis J. G. Langley, Jayden O. van Horik, Joah R. Madden

**Affiliations:** 1 Centre for Research in Animal Behaviour, Psychology, University of Exeter, Exeter, EX4 4QG UK; 2 School of Life Sciences, University of Lincoln, Lincoln, LN6 7DL UK

## Abstract

Brain lateralization is considered adaptive because it leads to behavioral biases and specializations that bring fitness benefits. Across species, strongly lateralized individuals perform better in specific behaviors likely to improve survival. What constrains continued exaggerated lateralization? We measured survival of pheasants, finding that individuals with stronger bias in their footedness had shorter life expectancies compared to individuals with weak biases. Consequently, weak, or no footedness provided the highest fitness benefits. If, as suggested, footedness is indicative of more general brain lateralization, this could explain why continued brain lateralization is constrained even though it may improve performance in specific behaviors.

## Introduction

Lateralization of the brain facilitates separate and parallel processing in the two hemispheres benefiting a lateralized individual^1^. Brain lateralization is commonly revealed by behavioral side biases, with more strongly biased individuals being more successful in specific, isolated cognitive and motor tasks e.g.^2–4^, more efficient foragers e.g.^5,6^, exhibiting more successful sexual displays^7^ and having more effective anti-predator behavior^8^. Despite these benefits, few, if any populations are uniformly and extremely lateralized. Instead, variance in both the direction and strength of lateralization and behavioral bias^9^ within populations is common. Strong selection pressures seem to favor brain and behavioral lateralization, yet lateralization does not reach fixation. It is not clear what constrains continued exaggeration of lateralization.

Ten-week-old captive-reared pheasants (*Phasianus colchicus*) exhibited high inter-individual variation in strength of their foot preferences in step-up tasks (see Methods). Footedness, limb preference, or more generically, differences in the use of limbs, is a commonly used indicator of brain lateralization in many vertebrate species, including galliformes, as it indicates a cerebral dominance of the contralateral hemisphere in motor control^1,10^. This may manifest in biasing a specific behavior (e.g. foraging or in our case, preference for a foot to step up onto a platform) or, more generally in associated motivational or emotional states^11^. The link between footedness and brain lateralization may be indirect, with asymmetries in limb use reflecting not only true motor laterality but also the outcome of other perceptual or cognitive processes which lead to a limb preference (e.g. eye preference leading to preferred limb use in cuttlefish^12^.

**Figure 1:**
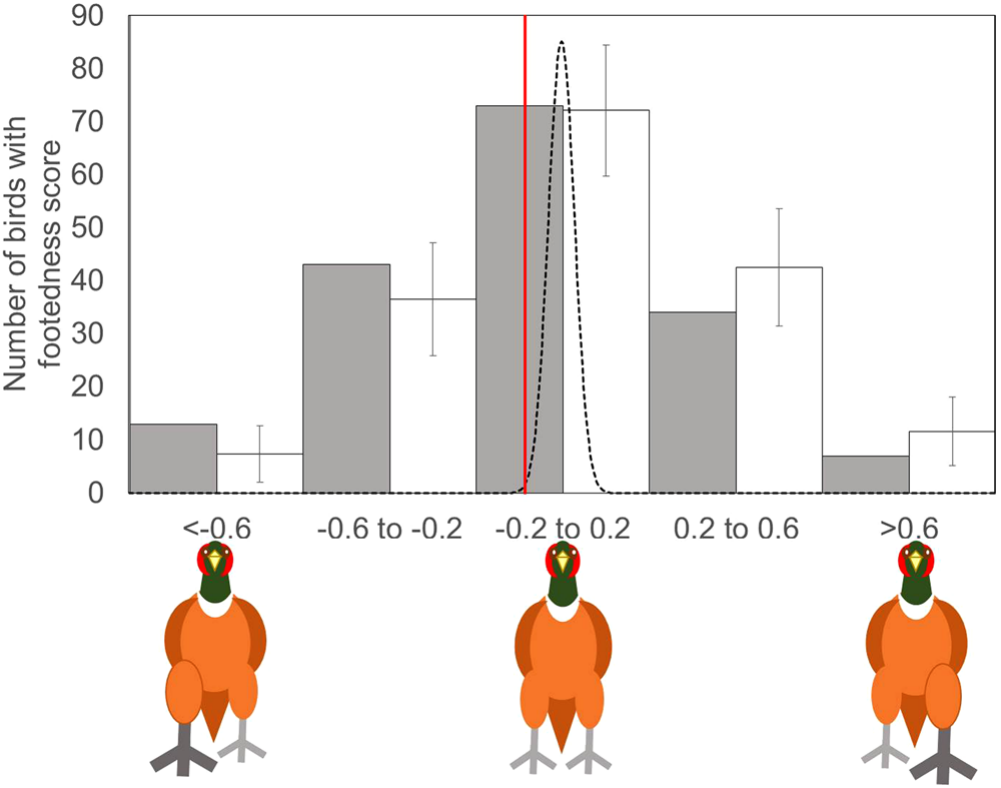
The strength and direction of footedness, measured as a laterality index based on the numbers of times an individual was observed using their left or right foot to step onto/over an obstacle (formula: (left − right)/(left + right)), for the observed population (grey bars) and an expected population that behaved randomly based on the number of times we watched a bird and that exhibited an even likelihood of using the right foot (white bars; error bars indicate ±1 SD). -1 represents an individual which has a right footed bias, an individual with +1 has a fully left footed bias and an individual with 0 has no bias. The solid red line indicates the mean strength of footedness (-0.101) for the tested population. This compares to a population subject to the same sampling regime but which was randomly footed (Samples >4 and released: mean = −0.00026, SD = 0.034, Randomization test: n = 5000, P = 0.004). The dashed line represents a normal distribution around −0.00026 of the expected population.

At a population level, there was a significant, albeit weak, right footed bias compared to distributions drawn from a sampling regime assuming random footedness (Fig. 1). Pheasants preferentially used their right foot about 10% more than would be expected by chance. Such population-level bias and high levels of intra-species variation is common in galliformes. Domestic chickens (*Gallus gallus*) and bobwhite quail (*Colinus virginianus*) also present a population-level right footedness, whereas Japanese quail (*Coturnic cortunix japonica*) have no population-level footedness and only weak individual foot preference^13^.

Pheasants have laterally placed eyes, eat an omnivorous diet and are both a predator and a prey species that live in groups^14^. In the wild, pheasants suffer from high mortality during early life, predominantly caused by starvation and predation^14,15^. Therefore, a lateralized brain in a pheasant could deliver fitness advantages through improved prey detection, predatory defense and group level movement. A strong footedness, arising from this lateralization, might lead to a turning bias and thus to coordinated group level movements or simply to a better and more efficient motor control when escaping from predators or when catching prey, leading to fitness advantages. Lateralization may also result in fitness benefits unrelated to movement influenced by footedness. In domestic chickens, non-lateralized chicks give more distress calls and take longer to resume foraging after exposure to a simulated predator than do lateralized chicks^1^.

Thus, further exaggerated lateralization appears to be favorable. However, the weak population-level bias and high variance in the strength of bias among individuals that we (and others [1, 8]) observed suggests that although some specific lateralized behaviors are favored, other tasks may not require strong lateralization. Here it is important to underline that lateralization manifests itself in a variety of behaviors and that limb preference is only one of them. Thus, in our study, the pheasants that showed strong footedness in the stepping task may have been weakly lateralized for other tasks such as, for example, eye use in detecting predators. Moreover, the extent of limb preference depends highly on the task^10,16^. Although strong handedness seems to improve feeding efficiency in chimpanzees^6^ and motor control in locusts^4^, where more strongly lateralized individuals outperform less lateralized conspecifics, this may not be the case for tasks such as stepping or standing. Indeed, standing or stepping are tasks which do not really require active or fine motor control compared to, for example, food reaching. Cats tested for multiple motor tasks display a significantly weaker lateral bias for lying side than in food reaching, step down or step over tests, in which cats show the same strength of paw preference^17^. Furthermore, some tasks demand the obligate use of the processing specialization of one hemisphere over the other, resulting in a stronger motor bias and, interestingly, this depends on the nature of the task rather than its complexity *per se*^11,18^. Macaques show a consistent hand preference when they select a preferred food and a non-preferred food, but not in a simple-reaching test^19^. This may explain why cats are more weakly lateralized for the spontaneous behavior of lying side^17^, but also why our pheasants show weak footedness in the stepping task as both tasks are not very demanding motor challenges.

**Figure 2:**
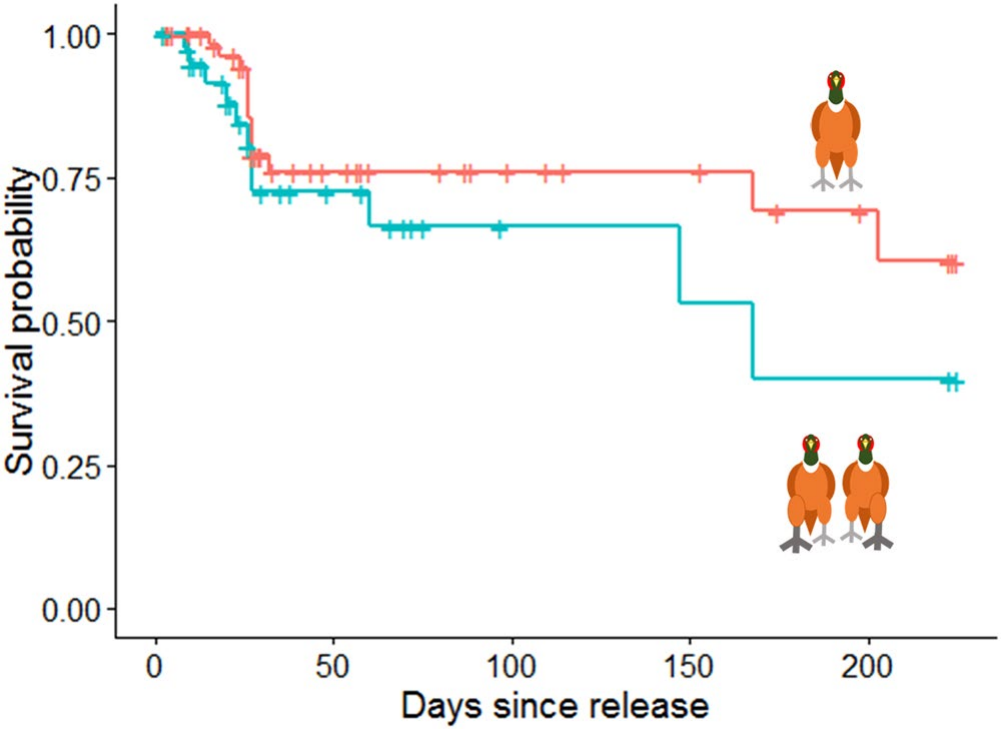
A Kaplan-Meier plot representing that the probability of survival for pheasants that were released with a lower than the mean bias in their footedness (N = 64, red line) or higher than the mean bias in footedness (N = 39, blue line). Footedness was recorded after the chicks were 16 days old and calculated regardless of directions (formula: √ ((left − right)/(left + right))^2^. +represents censored points.

We found evidence for stabilizing selection in our population, with individuals that exhibited stronger footedness bias early in life, regardless of direction (left or right), having a shorter life expectancy after release into the wild (see *Methods*) compared to individuals with weaker footedness bias (Regression coefficient = 1.77, Hazard ratio = 5.86, Lower 95% CI = 1.25, Upper 95% CI = 27.53, Χ^2^ = 4.38, *p* = 0.036, Fig. 2).

It was difficult to determine precisely the cause the death of all the pheasants. All retrieved carcasses showed evidence of predation. However, it is possible that these birds could have died from other causes, such as disease or starvation and their body had been subsequently scavenged. Therefore we cannot confidently discern the natural cause of death; however, the question still remains. Why, in pheasants, do we not see a survival benefit for individuals with higher degrees of footedness, given that other species-specific behaviors likely to improve survival are more effective when exhibited by more strongly biased/lateralized individuals^9^? A simple answer may be that strongly lateralized pheasants behave in ways which make them more susceptible to predation. Strong population-level lateral bias can make individuals more predictable to predators^8^. However, pheasants are predominantly predated by foxes and raptors^14^, that are ambush predators and unlikely to chase pheasants, thus predictability may not be a factor. An alternative constraint is that increased lateralization may retard other cognitive processes. Fish (*Brachyraphis episcopi*) with a strong turning bias are less able to navigate through a maze^20^. If this link between lateralization and spatial cognition manifests in pheasants then a pheasant with poor navigation would take a long time to find refuges or feeding sites and thus be susceptible to predation or starvation. A third constraint is that increased lateralization may promote risky behaviors. Strongly lateralized convict cichlids (*Amatitlania nigrofasciata*) are quicker to emerge from a refuge, indicative of boldness^21^. Bolder animals, including pheasants, often suffer from poor survival^22^. Finally, the degree of lateralization may not linearly associate with benefits. In humans there is an inverted U-shape relationship between degree of laterality and performance in word matching and face decision tasks^23^, suggesting that a moderately asymmetrical brain would provide the greatest advantage. Consequently, there may be an optimum degree of laterality that returns the highest fitness benefits.

Using a system that requires the release and recovery of a large number of animals that had previously had their footedness scored provides a novel insight into the selection pressures that may act on laterality, however, it is not without its limitations. We meticulously searched the release site for carcasses and although we are confident in determining a date of death to within a few days, cold searching has the potential to introduce errors. Pheasants are large and easily detected but if location of death was biased by footedness (an extreme example: more strongly footed birds crawled into dense undergrowth to die) we may find that our search strategies biased the finding certain types of carcasses. Because most birds were killed by predators, particularly foxes, such bias could only occur if the predators themselves treated the carcasses differently according to the footedness of the dead bird. We suspect that this is unlikely. Improved precision and accuracy of detection of deaths could be achieved by continuous radio tracking of the entire released population.

It appears, at least in pheasants, that free-living individuals subject to a range of selection pressures including predation, starvation and disease have shorter life expectancies if they exhibit stronger footedness, suggesting greater brain lateralization. This could explain why we observe low, but persistent population-level footedness, even though more biased individuals may perform better in certain specific tasks that contribute to their survival. Although both genetic and environmental factors can determine lateralization^1^, multiple factors may contribute to generating the high initial variance that we and others have detected. We demonstrate that mortality may then sift this variance, penalizing more extreme expressions of lateralization and bias, and so reduce the variance in those survivors that breed, such that further exaggeration is prevented and lateralization is stabilized.

## Methods

### The rearing system

For 10 weeks we reared individually marked pheasants from one day old in four identical aviaries at North Wyke Farm, Devon, UK. For the first two weeks of life chicks had access to a heated house (2 m × 2 m). During the following weeks they had further access to a covered outdoor grass run (4 m × 12 m). Connected to the rearing house, and separated by a sliding door, was a testing arena (0.75 m × 0.75 m). We provided food and water *ad lib* and in excess alongside opportunities for perching.

### Measures of footedness

We assessed footedness by recording the leg an individual used to step up onto an obstacle. When the chicks were 16 days old we trained the birds to enter a chamber where they could be tested in isolation on cognitive tasks as part of a separate study. Upon exiting the testing chamber, into the outside runs, we placed a block (0.25 m × 0.05 m × 0.05 m) in front of the exit gate (that was placed centrally and directly opposite any testing apparatus in order to not introduce possible position biases) and recorded the foot that each bird used to step onto/over the block. We only considered instances when birds were stationary prior to stepping to remove the effect of gait on foot choice. We concentrated on observations after 16 days because, in chickens less than 11 days old^24^, the preferred leg was not consistent. We wanted a measure of footedness that best represented the behaviour of juveniles when they were released into the wild.

Footedness was measured for those birds that were observed stepping up more than four times by calculating their laterality index i.e. an index score defined by the formula: (left − right)/(left + right). A laterality index of 0 corresponded to individuals with no foot preference, whereas an index of 1 and −1 corresponded to individuals which exclusively used their left or right foot respectively. Of the 243 birds observed, 56 were used in a different experiment and so excluded from this study. A further 74 were observed <4 times and so we excluded these to reduce the chance of bias due to small samples. 10 birds were not released, so we could not follow their fate in the wild and they were excluded from our analyses.

Even for those birds observed >4 times, each bird was not observed the same amount of times (mean = 10; range = 5–26) and we suspected that small samples were more likely to bias an individual’s laterality index, therefore we used a randomization approach to test whether the strength and direction of footedness differed from a population that behaved randomly. We generated 5,000 random observation datasets for each bird based on the number of actual observations that we conducted on them. The number of right steps that they were assumed to take at random were drawn from a binomial distribution with a 50% chance of taking a step with their right foot. The calculations were made in Excel using the function ‘ = binom.inv([number of observations], 0.5, rand())’.

### Fate

At 10 weeks of age, all birds were weighed using a Samson spring balance (precision 0.5 g) and a Passive Integrated Transponder (PIT) ring was attached. They were initially mixed together and placed into a large release pen (~3000 m^2^). The pen was surrounded by a 2 m high fence which excluded terrestrial predators, and contained water and food, woodland, open grassland and canopy suitable for roosting. The pen was unroofed which meant they were susceptible to aerial predation but it also allowed birds to disperse freely onto the farm where they became susceptible to terrestrial predators such as foxes. On the farm we placed 40 wheat-dispensing feeders (0.16 per hectare), each monitored with a PIT tag reader and a motion sensitive camera that recorded individual feeding visits.

Fate of the pheasants after release into the wild was measured for 223 days, stopping on the 1^st^ March. We did this in three ways: 1) we conducted sweeps of the local site and recorded birds that were observed alive as well as birds that were dead, with their fate and time of death determined on site using field signs. For the first 30 days sweeps were conducted every day, thereafter sweeps were conducted twice a week until the end of the study. Pheasants are large and carcasses can easily be spotted and retrieved, with such meticulous coverage of the land we were confident that birds died between two visits to the site. We conservatively assigned the date of death as the day that we found the carcass. We recorded the maximum date for analysis; 2) we monitored feeder use via PIT tag or by analysing the photos taken by the motion sensitive camera, with detected birds being classed as alive; and 3) between 1 March 2017 and 30 March 2017 we conducted a systematic trapping regime using funnel traps baited with wheat. We checked these traps three times a day and recorded all individuals that were caught. We considered all birds caught in March to have been alive on 1 March 2017. We stopped measuring the fate of birds on 1^st^ March because the trapped birds were then brought into captivity for a separate study.

### Statistical analysis

We used a Cox’s proportional hazards model to assess if life expectancy was predicted by sex, release mass, strength of footedness, direction of footedness, and the number of observations used to determine the footedness bias. Survival analysis is used in many medical and public health studies where individuals have a precisely determined time of death (in our case measured by finding carcasses)^25^. The method can deal with cases when we do not know the timing of an event, for instance when we have unobserved deaths after the end of the study as well as accounting for presumed deaths occurring within the study period, which can be censored based on the time of last sighting^25,26^. For individuals that were found dead (n = 24) during the period of the study we allocated these with a censored value of 1. For individuals that we presumed died during the study period we censored at the time of last sighting with a value of 0 (n = 69). Finally, individuals that were definitely alive at the end of the study were also censored with a value of 0 (n = 10). We used a non-parametric model because our footedness data were uneven with a small number of extremely strongly footed individuals (see Fig. 1). A bird was categorised as having a right bias if they had a laterality index of less than 0, a left bias if they had a laterality bias more than 0 and no bias if their index equalled 0. In the survival model we initially included strength of footedness, the number of observations used to determine footedness, the direction of footedness (left or right) and mass upon release as main effects. We also added the interaction between sex and strength of footedness. The model was simplified using a backward stepwise deletion of non-significant terms based on likelihood ratios until variables could not be removed without increasing the variance, allowing us to report the predictors that best represent the data collected. We only reported the results of the minimum adequate model. Birds that were included in the model were all birds that were released into the wild and which were observed five or more times during early development to establish the scores for the laterality index. Analyses were conducted in R^27^ using the package survival^28^.

### Ethical statement

All work was approved by the University of Exeter Psychology Ethics Committee and conducted under Home Office licence number PPL 30/3204. All methods were performed in accordance to relevant guidelines and regulations.

## Data Availability

The datasets generated analyzed during the current study are available from the corresponding author on reasonable request.
